# Microstructure and Impact Toughness of Local-Dry Keyhole Tungsten Inert Gas Welded Joints

**DOI:** 10.3390/ma12101638

**Published:** 2019-05-20

**Authors:** Shuwan Cui, Zhiyong Xian, Yonghua Shi, Baoyi Liao, Tao Zhu

**Affiliations:** 1 School of Mechanical and Automotive Engineering, South China University of Technology, Guangzhou 510640, China; 13597066615@163.com (S.C.); 13430279851@163.com (B.L.); zt20090609@163.com (T.Z.); 2 Guangdong Provincial Engineering Research Center for Special Welding Technology and Equipment, South China University of Technology, Guangzhou 510640, China; 3 Foshan Supervision Testing Center of Quality and Metrology, Foshan 528225, Guangdong, China; Xianzhiyong@126.com

**Keywords:** underwater local-dry keyhole tungsten inert gas welding, impact toughness, phase interface, Σ3 coincidence site lattice boundary

## Abstract

In this paper, the microstructure and impact toughness of a S32101 duplex stainless steel underwater local-dry keyhole tungsten inert gas welded joint were studied. The impact toughness value of the underwater weld metal reached 78% of the onshore weld metal, which is in accordance with the underwater welding standards. The proportion of austenite in the underwater weld metal was 0.9% lower than that of the onshore weld metal. The proportion of the Σ3 coincidence site lattice boundaries and random phase boundaries in the underwater weld metal, which significantly influence the impact toughness of the weld metal, were smaller than that of the onshore weld metal.

## 1. Introduction

Duplex stainless steel (DSS) is a structural and functional integrated material that possesses high strength and outstanding corrosion resistance. DSS combines many advantages of austenite and ferrite stainless steel; thus, it is widely used in petroleum and chemical and ocean engineering, as reported by Shen et al. [1]. Guo et al. [2] pointed out that DSS has become a hot topic in research of new ferrous materials. Mourada et al. [3] used gas tungsten arc and laser beam welding methods to study DSS, while Shi et al. [4] also studied DSS but used the keyhole tungsten inert gas (K-TIG) welding method.

Underwater welding can be divided into three methods: Dry welding, wet welding and underwater local cavity welding [5]. Dry welding is usually performed in a high-pressure chamber underwater and can obtain a high-quality welded joint, but the welding equipment of dry welding is very complicated and the welding cost is high, as reported by Shi et al. [6]. The cooling rate in wet welding is relatively high, which can lead to a remarkable decrease in the mechanical properties of the welded joints. In addition, Guo et al. [7] proved that under the effect of water and pressure, wet welded joints are prone to defects such as blowholes and cracks. By contrast, local-dry welding can eliminate the effect of water on the surface of the weld bead and ensure the quality of the welded joint. Therefore, local-dry welding is an ideal method for underwater welding.

Currently, studies focus on traditional underwater local-dry welding methods, such as local-dry tungsten inert gas welding and local-dry gas metal arc welding. These welding methods generally require multiple passes to complete the welding of plates more than 3 mm in thickness. In the DSS multi-pass welding procedure, precipitation of harmful phases, such as sigma and carbides, leads to the formation of a chromium-depleted area that can decrease the quality of the underwater welded joint. In addition, the underwater welding environment is very harsh. Therefore, longer welding times are less favorable and the development of efficient and time-saving underwater welding technology is a key focus in underwater welding research.

Cui et al. [8,9] have successfully welded 10.8 mm DSS plates and have proven that the mechanical and intergranular corrosion properties of K-TIG welded joints are excellent. At present, K-TIG welding has attracted the attention of the research community [10,11]; however, no researchers have applied K-TIG welding to underwater welding. Underwater local-dry K-TIG (ULK-TIG) welding is a new type of underwater welding method that can reconstruct and repair large underwater structures. Based on K-TIG welding, ULK-TIG welding added a local-dry cavity that can exclude water in the weld and arc in order to obtain a high-quality welded joint during the welding procedure. ULK-TIG welding has the characteristics of a non-filling metal and strong penetration ability, and it can save welding materials and reduce costs to a certain extent. However, the microstructure of ULK-TIG welded joints have not been studied and it is also unclear whether the impact toughness of an ULK-TIG welded joint meets the requirements of the mechanical properties of underwater welding. Therefore, it is highly significant to study the microstructure and impact toughness of ULK-TIG welded joints.

## 2. Materials and Experimental Procedure

**Figure 1:**
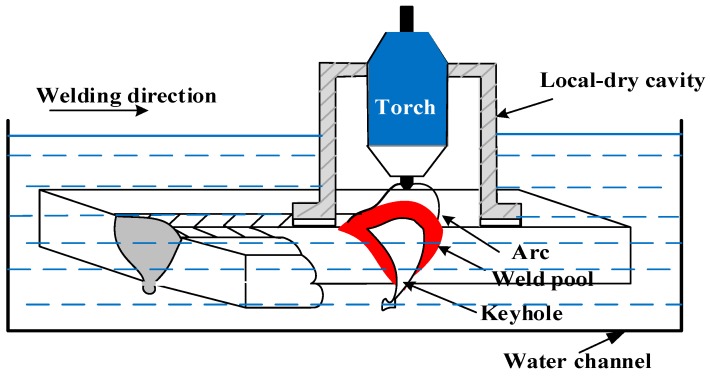
The underwater local-dry (UL) K-TIG welding equipment.

Steel used in the experiment was S32101 DSS, which served as the base metal (BM). The BM composition is listed in Table 1. The edges of 300 × 100 × 10.5 mm BM samples were welded by using an ULK-TIG welding system with a welding current and welding speed of 460 A and 210 mm/s, respectively. The DSS plates were mechanically cleaned by a polisher, and the groove was not prepared before ULK-TIG welding. The welding parameters were selected according to previous studies and are listed in Table 2 [8]. A detailed description of the ULK-TIG welding equipment is shown in Figure 1.

**Figure 2:**
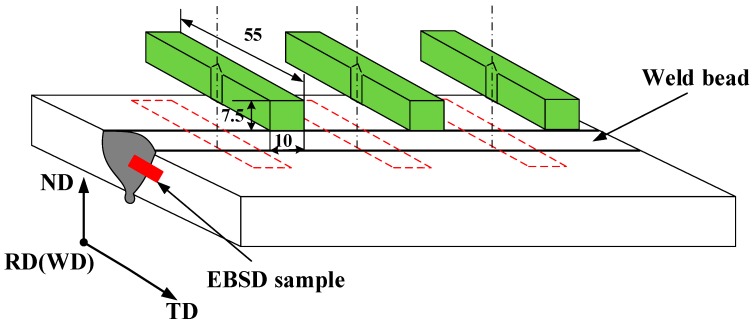
Dimensions of the test specimens (units: mm).

After ULK-TIG welding, Charpy impact tests were performed at room temperature according to the ASTM A370 [12]. The Charpy test specimens, with a thickness of 7.5 mm, were used to detect the impact toughness of the BM and weld metal (WM) in the ULK-TIG welded joint. A schematic of a Charpy impact specimen is displayed in Figure 2. In the figure, ND, TD and RD represent the normal direction, transverse direction and rolling direction, respectively. The specimens were polished and etched before the V-notch was performed, with the V-notch center located on the WM. BM and WM impact tests in the ULK-TIG welded joint were repeated three times. The fracture morphologies of the BM and WM were examined by SEM, providing insight into the fracture method of the BM and WM.

An optical microscope (OM) was used to observe the microstructure of the ULK-TIG welded joint that was etched by a Beraha etchant. Crystallographic information on the ULK-TIG welded joint, including the texture of the austenite, the phase interface between ferrite and austenite, and the grain boundary misorientation-angle distribution (GBMAD) of the BM, heat-affected zone (HAZ), and WM, was characterized by an electron backscatter diffraction (EBSD) detector attached to the scanning electron microscope. The EBSD specimens were electrolytically polished with 10 vol.% perchloric acid, 60 vol.% butyl alcohol and 30 vol.% methyl alcohol solution at −20 °C. The accelerating voltage was 20 kV, distance from the sample to the EBSD detector was 5 mm and step size was 1.5–3.5 μm in the EBSD data acquisition process. Channel 5 software was used to perform the postprocessing steps and the analysis of the collected EBSD data.

## 3. Results and Discussion

### 3.1. Impact Toughness of the ULK-TIG Welded Joint

**Figure 3:**
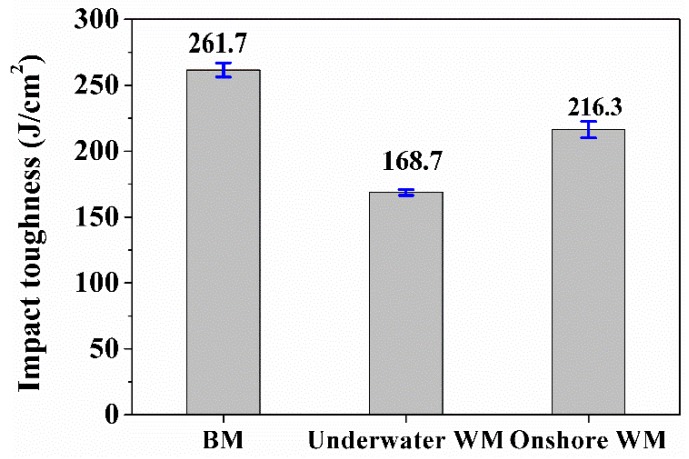
Impact toughness of the BM, underwater weld metal (WM) and onshore WM.

During the ULK-TIG welding process, the cooling rate of the HAZ was relatively high and the width of the HAZ in the ULK-TIG welded joint was always less than 1 mm. Therefore, it was difficult to test the impact toughness of the HAZ. The impact toughness values of the BM and WM in the ULK-TIG welded joint are shown in Figure 3. To compare the impact toughness of ULK-TIG WM (underwater WM) with the impact toughness of onshore K-TIG WM (onshore WM), the impact toughness of onshore WM was obtained from previous research [13], and is also presented in Figure 3. The mean values of impact toughness in the BM and underwater WM were 261.7 J/cm^2^ and 168.7 J/cm^2^, respectively, while the mean value of impact toughness for onshore WM was 216.3 J/cm^2^. The standard deviations of impact toughness in the BM, underwater WM and onshore WM were 5.4, 2.2 and 6.2, respectively. The impact test results indicate that the impact toughness value of the underwater WM reaches 64.5% of the BM and 78% of the onshore WM, which is in accordance with the underwater welding standards. 

**Figure 4:**
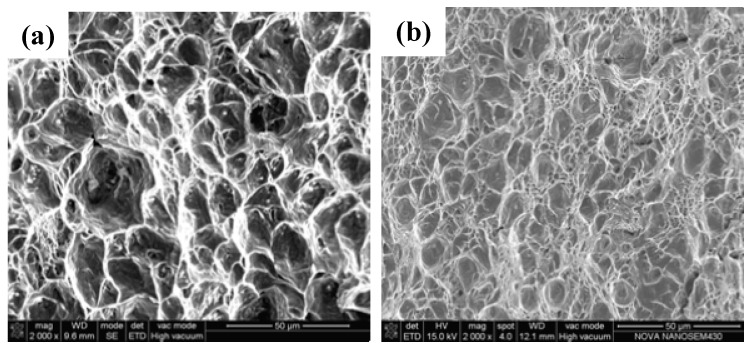

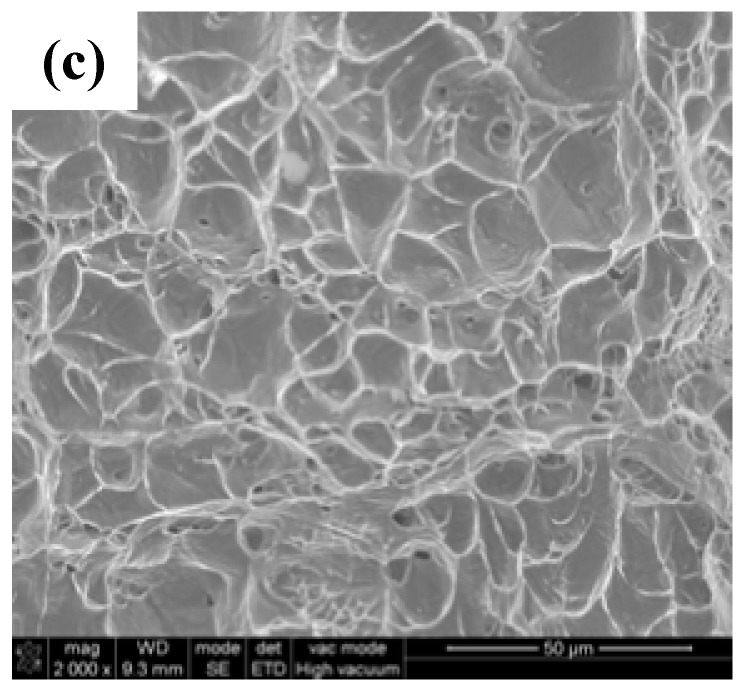
SEM fracture surfaces of the ULK-TIG welded joint after Charpy impact tests. (**a**) BM, (**b**) underwater WM and (**c**) onshore WM [13].

The fracture surfaces of the BM, underwater WM and onshore WM are shown in Figure 4. The fracture surfaces of the BM and onshore WM indicate a ductile fracture feature with a large number of dimples, implying that the BM and onshore WM undergo severe plastic deformation before fracturing. Figure 4b displays the fracture surface of the underwater WM. Although the value of the impact toughness in the underwater WM was lower than that of the BM and onshore WM, a large number of dimples were observed at the fracture surface of the underwater WM, which also demonstrates that it undergoes a ductile fracture. Under the same magnification, comparison of the SEM fracture micrographs of the BM, underwater WM and onshore WM shows that the mean sizes of the dimples in the BM and onshore WM are larger than that of the underwater WM. This indicates that the BM and onshore WM undergo greater plastic deformation and absorb more energy before fracturing. Thus, with reference to the SEM fracture micrographs, the impact toughness of the BM and onshore WM are better than that of the underwater WM. Because the cooling rates of ULK-TIG welding and onshore K-TIG welding are different, the microstructure of the underwater WM and onshore WM are also different, which may be the reason for the effects observed for the impact toughness of the WM. Therefore, it is necessary to compare the microstructure of the underwater WM with that of the onshore WM.

### 3.2. Microstructure and Austenite Phase Proportion

**Figure 5:**
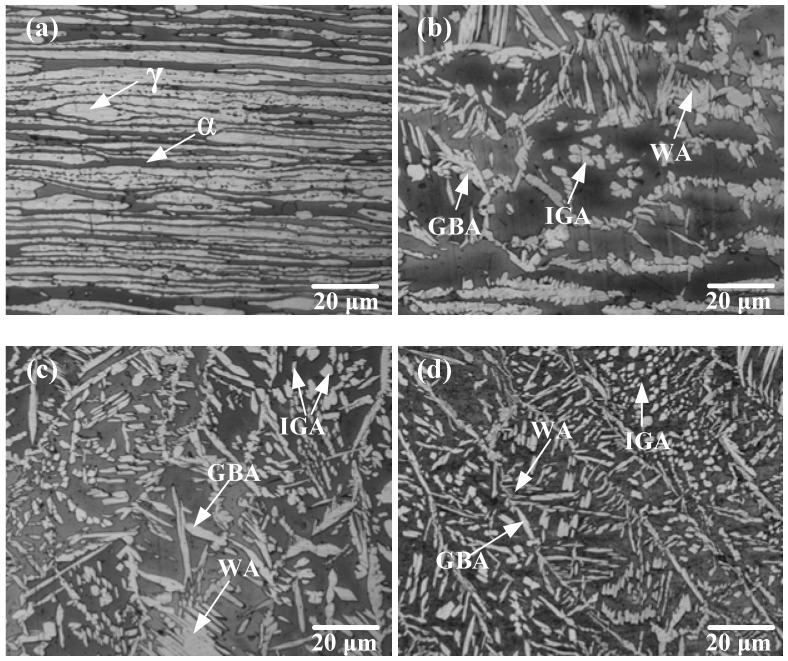
The microstructure of the ULK-TIG welded joint and onshore WM: (**a**) BM, (**b**) heat-affected zone (HAZ), (**c**) underwater WM and (**d**) onshore WM.

The microstructure of the ULK-TIG welded joint was observed using an OM, as shown in Figure 5a–c. The BM was a rolled material composed of 51.9% austenite (γ) phase and 48.1% ferrite (α) phase. Figure 5a clearly shows that the distribution of the γ phase and α phase in the BM was relatively uniform. In the HAZ, the austenite was deformed and some austenite was dissolved in ferrite due to the high temperature, which coarsened the ferrite grains. Most of the austenite precipitated as grain boundary austenite (GBA) and Widmanstätten austenite (WA) during the subsequent cooling process, as shown in Figure 5b. Figure 5c shows that the austenite in the WA is found mainly in the form of intergranular austenite (IGA), GBA and WA. Most of the WA laths were broken from the middle, but were not yet fully converted into independent IGA. There were no intermetallic phases in the underwater WM. The microstructure of the onshore WM is shown in Figure 5d. Compared with the underwater WM, the microstructure of the onshore WM contains a greater amount of IGA.

**Figure 6:**
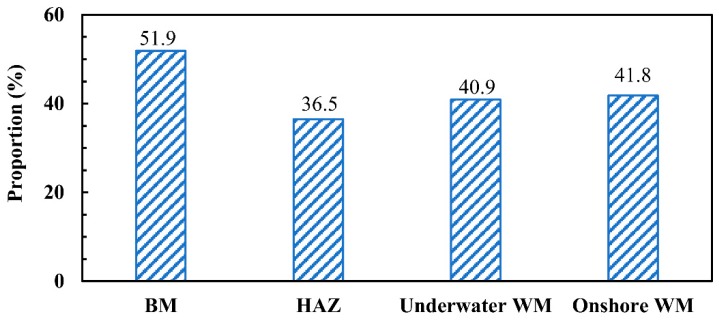
The phase proportions of the austenite in the ULK-TIG welded joint and onshore WM.

Zhang et al. [14] found that the phase proportion of the austenite in DSS materials can affect the impact property of the WM. When the phase proportion of the austenite increases, the value of the impact toughness in the WM increases. The phase proportions of the austenite in the ULK-TIG welded joint are shown in Figure 6a–c, and the phase proportion of the austenite in the onshore WM is shown in Figure 6d. It may be observed that the phase proportions of the austenite in the BM, HAZ, underwater WM and onshore WM are 51.9%, 36.5%, 40.9% and 41.8%, respectively. It is clear that the phase proportion of the austenite in the underwater WM is closer to that of the onshore WM. However, as shown in Figure 3, the impact toughness of the underwater WM is quite different from that of the onshore WM. This proves that the phase proportion of the austenite does not have a strong influence on the impact toughness of the WM. 

### 3.3. Phase Boundary Characteristics and GBMAD

Zhang et al. [15] reported that during the deformation process, the characteristics of the phase interface between ferrite and austenite play a significant role and affect the impact toughness of the DSS. Generally, based on the Kurdjumov–Sachs (K–S) model, the orientation of the phase interfaces between ferrite and austenite are coherent or semicoherent, while the other phase interfaces are incoherent interfaces. Zhang et al. [16] indicated that the exact K–S orientation relationship almost does not exist. Therefore, according to the calculated deviations from the exact K–S orientation, the phase boundaries of austenite and ferrite in DSS are divided into two types. Karlsson and Börjesson [17] revealed that the phase boundaries close to K–S orientation (within 6°) are special phase boundaries (SPB) and the phase boundaries with angles larger than 6° are random phase boundaries (RPB). 

**Figure 7:**
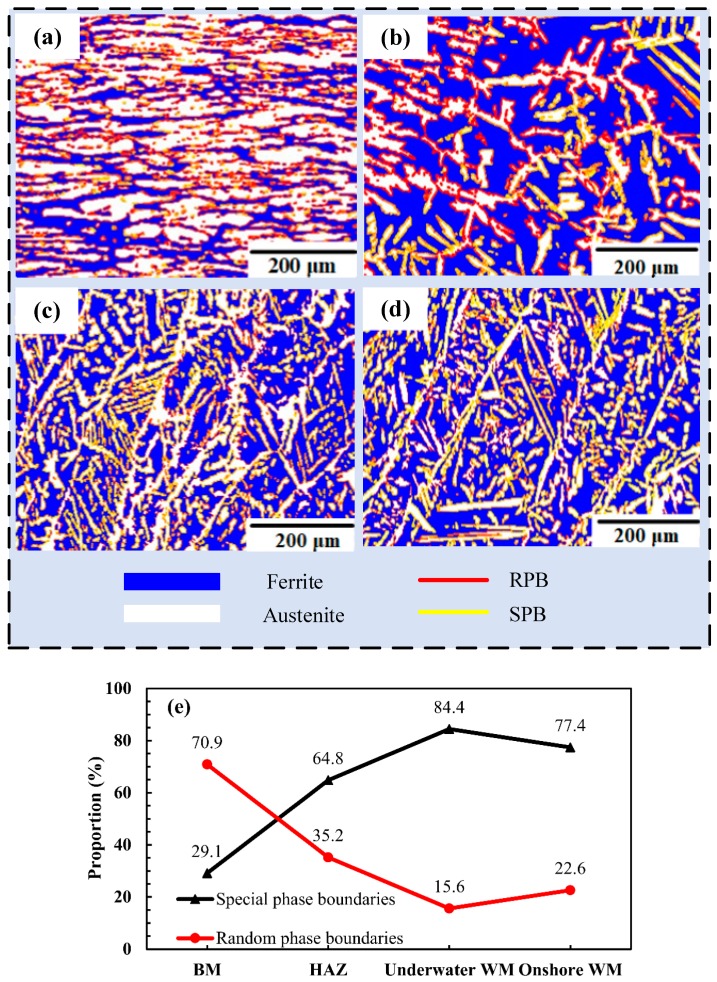
The orientations of the phase interfaces between ferrite and austenite: (**a**) BM, (**b**) HAZ, (**c**) underwater WM, (**d**) onshore WM and (**e**) The proportion of special phase boundaries (SPB) and random phase boundaries (RPB).

Figure 7a–c shows the orientation of the phase interfaces between ferrite and austenite in the ULK-TIG welded joint, while Figure 7d shows the orientation of the phase interfaces between ferrite and austenite in the onshore WM. In this figure, the blue phase is ferrite, the white phase is austenite, the yellow lines represent SPB and the red lines represent RPB. The proportions of SPB and RPB in the BM, HAZ, underwater WM and onshore WM are shown in Figure 7e. 

It can be observed that the proportion of SPB in the BM, HAZ, underwater WM and onshore WM is 29.1%, 64.8%, 84.4% and 77.4%, respectively. The proportion of RPB in the BM, HAZ, underwater WM and onshore WM is 70.9%, 35.2%, 15.6% and 22.6%, respectively. Patra et al. (2016) [18] showed that stress accumulation at an incoherent interface was more easily released by sliding at the interphase boundary. Zhang et al. [15] also reported that incoherent interfaces are more prone to slip than coherent interfaces. It can be inferred that a higher proportion of RPB facilitates the release of the sliding interface boundary. When the proportion of RPB is large, the impact toughness of the material is better. Figure 7 shows that the proportion of RPB in the BM is the largest and the proportion of RPB in the underwater WM is lower than that of the onshore WM. Analysis of the phase boundary results prove that the impact toughness of the underwater WM is lower than that of the onshore WM, which is consistent with the impact test results. Therefore, it can be expected that RPB have an effect on the impact toughness of the materials. The impact toughness of the WM increases with an increasing proportion of RPB.

### 3.4. The GBMAD of the Austenite and Ferrite

GBMAD has a strong influence on the impact toughness of polycrystalline materials. Based on their orientation angle (θ), the grain boundaries were divided into two types: Low-angle grain boundaries (LAGB) and high-angle grain boundaries (HAGB). The θ of the LAGB was 2–10° and the θ of the HAGB was greater than 15°, as described by Park et al. [19]. Both Michiuchi and Wang [20] and Jones and Randle [21] reported that when the θ of a grain boundary is 60°, it is called a Σ3 coincidence site lattice (CSL) grain boundary. This is a special type of grain boundary that possesses low-energy and low-impurity segregation. A Σ3 CSL boundary has stronger resistance to fracture than other grain boundaries. Therefore, during the process of material deformation, the frequency of Σ3 CSL grain boundaries will influence the impact toughness of the material.

**Figure 8:**
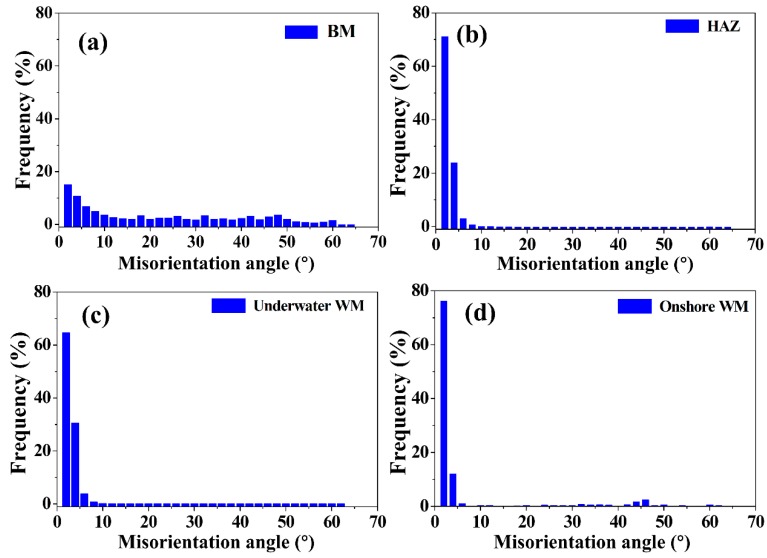
Grain boundary misorientation-angle distribution (GBMAD) of ferrite for the (**a**) BM, (**b**) HAZ, (**c**) underwater WM, and (**d**) onshore WM.

**Figure 9:**
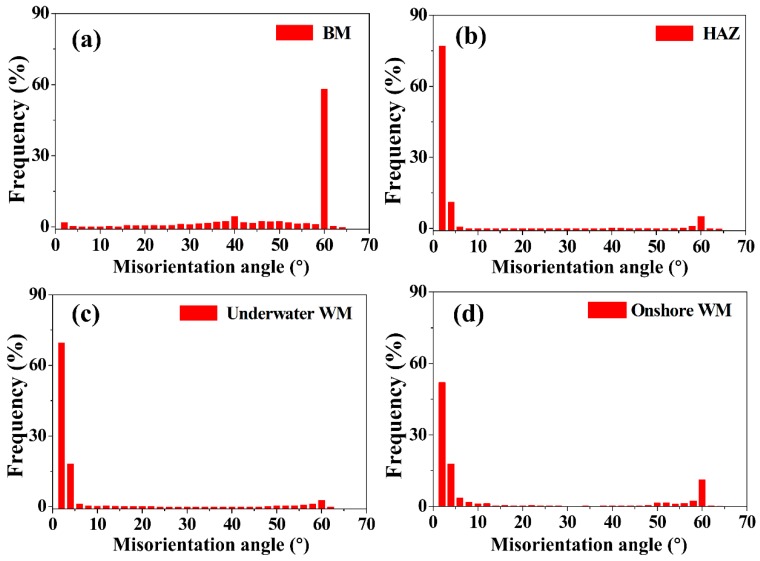
GBMAD of austenite for the (**a**) BM, (**b**) HAZ, (**c**) underwater WM, and (**d**) onshore WM.

The GBMAD of ferrite in the BM, HAZ, underwater WM and onshore WM is shown in Figure 8. The frequency of LAGB and HAGB in the ferrite of the BM was almost equal. However, the GBMAD of ferrite in the HAZ, underwater WM and onshore WM was mainly distributed in LAGB. This is in contrast with Figure 9, which shows the GBMAD of austenite in the BM, HAZ, underwater WM and onshore WM, wherein it can be clearly seen that Σ3 CSL boundaries mainly appear in the austenite grains. 

**Figure 10:**
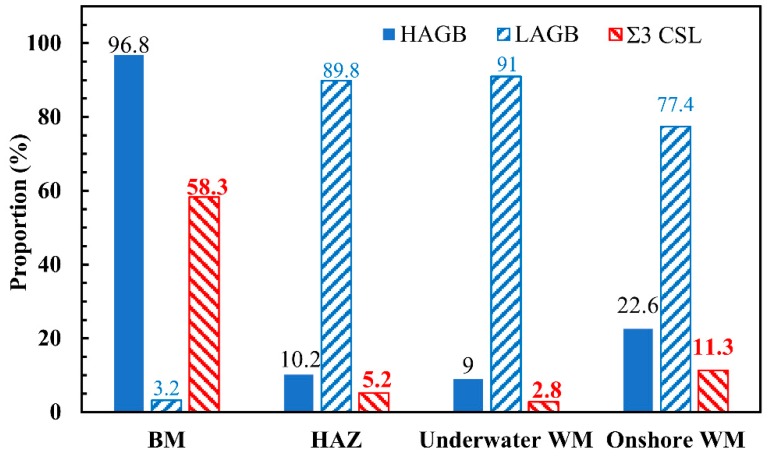
The proportion of high-angle grain boundaries (HAGB), low-angle grain boundaries (LAGB) and Σ3 coincidence site lattice (CSL) boundaries for austenite in the BM, HAZ, underwater WM and onshore WM.

Figure 10 shows the proportion of HAGB, LAGB and Σ3 CSL boundaries in the austenite of the ULK-TIG welded joint and the onshore WM. The frequency of Σ3 CSL boundaries in the austenite of the BM was 58.3%, which was higher than that of the HAZ, underwater WM and onshore WM. The GBMAD of austenite in the BM was mainly distributed in HAGB. However, the GBMADs of austenite in the HAZ, underwater WM and onshore WM were mainly distributed in LAGB. The proportion of Σ3 CSL boundaries in the austenite of the HAZ, underwater WM and onshore WM was reduced to 5.2%, 2.8% and 11.3%, respectively. The proportion of Σ3 CSL boundaries in the underwater WM was higher than that of the onshore WM. Σ3 CSL boundaries have the best fracture resistance among all the grain boundary types. Together with the results of the impact test, these findings suggest that a decrease in the proportion of Σ3 CSL boundaries can also reduce the impact toughness of the materials. Therefore, the proportion of Σ3 CSL boundaries has a significant influence on the impact toughness of the WM. 

## 4. Conclusions

(1). The ULK-TIG welding system successfully welded S32101 DSS (10.5 mm) in a single pass without filling metals and without groove preparation.
(2). The impact toughness value of the underwater WM reaches 64.5% of the BM and 78% of the onshore WM, which is in accordance with underwater welding standards.
(3). The proportion of the phase, RPB and Σ3 CSL boundaries influenced the impact toughness of the WM.

## Figures and Tables

**Table 1:** Chemical composition of the base metal (BM) (wt.%).

Element	Cr	Ni	Mn	Mo	Cu	Si	C	N	S	P	Fe
wt%	21.52	1.56	4.98	0.22	0.16	0.49	0.017	0.21	0.002	0.02	Bal.

**Table 2:** Welding parameters used during the underwater local-dry keyhole tungsten inert gas (ULK-TIG) welding experiments.

Weld current	460 A
Arc voltage	17.1 V
Weld speed	210 mm/s
Diameter of tungsten electrode	6.4 mm
Electrode gap	2.5 mm
Shielding gas	Pure argon (99.9%)
Gas flow rate	20 L/min
